# Device indication for calcified coronary lesions based on coronary imaging findings

**DOI:** 10.1007/s12928-025-01179-6

**Published:** 2025-08-22

**Authors:** Yuji Ikari, Teruyasu Sugano, Nobuhiko Ogata, Shinjo Sonoda, Kazuhiko Nakazato, Junya Ako, Toshiro Shinke, Yoshio Kobayashi, Ken Kozuma

**Affiliations:** 1 Department of Cardiology, Tokai University, Isehara, Japan; 2 Department of Cardiology, Saiseikai Yokohamashi Nanbu Hospital, Yokohama, Japan; 3 Department of Cardiology, Ageo Central General Hospital, Ageo, Japan; 4 Department of Cardiology, Japanese Red Cross Karatsu Hospital, Karatsu, Japan; 5 Department of Cardiovascular Medicine, Fukushima Medical University, Fukushima, Japan; 6 Department of Cardiovascular Medicine, Kitasato University, Sagamihara, Japan; 7 Department of Cardiology, Showa Medical University, Shinagawa, Japan; 8 Department of Cardiovascular Medicine, Chiba University Graduate School of Medicine, Chiba, Japan; 9 Department of Cardiology, Teikyo University Hospital, Itabashi, Japan

**Keywords:** Rotational atherectomy, Intravascular lithotripsy, Orbital atherectomy, Intravascular ultrasound, Optical coherence tomography, Optical frequency domain imaging

## Abstract

Performing percutaneous coronary intervention (PCI) for heavily calcified coronary lesions remains a significant clinical challenge. In 2023, following the availability of intravascular lithotripsy (IVL), a consensus document was published outlining imaging-guided device selection strategies for the treatment of calcified lesions. Since the publication of that document, the DUAL-PREP study has demonstrated the safety of combining rotational atherectomy (rotablator) with IVL, a strategy previously contraindicated in the original consensus. As a result, a revision of the consensus document became necessary. In the updated consensus, the fundamental principle of imaging-guided treatment planning is retained. However, a key modification is the acknowledgment that IVL may now be considered in cases where post-atherectomy imaging reveals persistent heavy calcification and further atherectomy is deemed either ineffective or potentially harmful to the patient.

## Introduction

Performing percutaneous coronary intervention (PCI) for heavily calcified lesions remains a significant clinical challenge. The presence of calcification often results in suboptimal lesion dilatation, which is associated with both poor short-term procedural success and unfavorable long-term outcomes. Pre-stent modification of calcified plaques has been proposed as a strategy to facilitate adequate expansion and thereby improve clinical results. In 2023, with the advent of intravascular lithotripsy (IVL), a consensus document was published outlining an imaging-based algorithm for device selection in the treatment of calcified coronary lesions [1]. Subsequent to this publication, the DUAL-PREP study demonstrated the safety of combining rotational atherectomy with IVL [2]. As this combination had been contraindicated in the previous consensus, an update to the recommendations became necessary.

### Device selection strategy for calcified coronary lesions

**Fig. 1:**
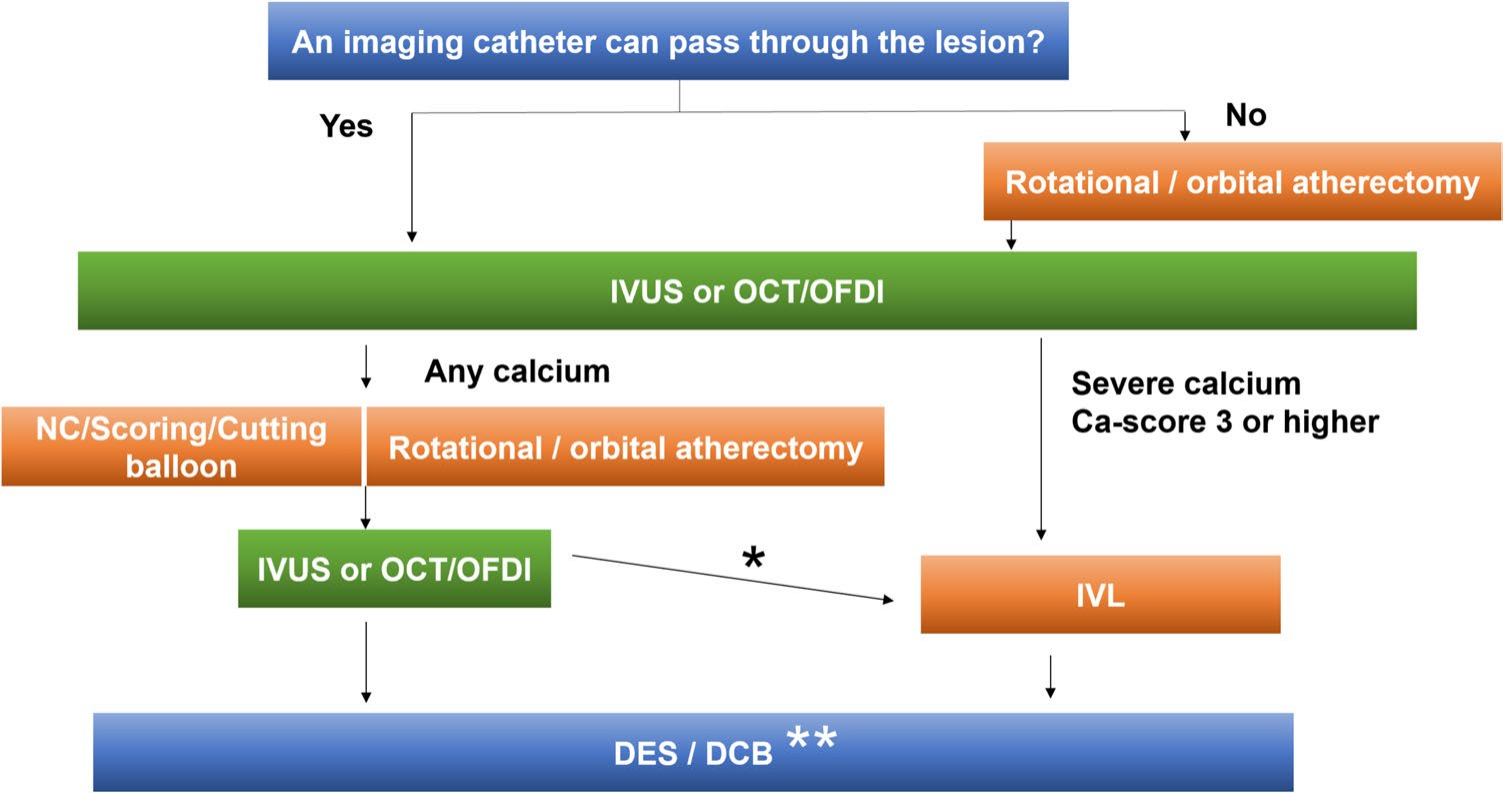
Device selection strategy for calcified lesions. *The criteria for IVL use after atherectomy are as follows: In addition to meeting criterion (1), the case must also satisfy either criterion (2) or (3): (1) Post-atherectomy imaging reveals a residual calcium score ≥ 3. (2) Further atherectomy is contraindicated due to clinical factors such as reduced left ventricular function or impaired renal function. (3) Additional atherectomy is unlikely to be effective based on lesion characteristics. **DES use is generally recommended after IVL use. *IVUS* intravascular ultrasound, *OCT* optical coherence tomography, *OFDI* optical frequency domain imaging, *NC* non-compliant, *IVL* intravascular lithotripsy, *DES* drug-eluting stent, *DCB* drug coated balloon
- **Step 2:** If the imaging catheter successfully crosses the lesion, the severity of calcification should be evaluated. Table 1 outlines the criteria for assessing calcium severity using IVUS or OCT/OFDI. If the imaging catheter fails to cross the lesion, plaque modification with rotational or orbital atherectomy should be considered.

- **Step 1:** following successful guidewire crossing, the first step is to attempt lesion crossing with an intravascular imaging device such as intravascular ultrasound (IVUS) or optical coherence tomography/optical frequency domain imaging (OCT/OFDI) (Fig. 1).

**Table 1:** Calcium scoring by OCT/OFDI or by IVUS

OCT/OFDI	Points
Maximum calcium angle > 180°	2
Maximum calcium thickness > 0.5 mm	1
Calcium length > 5 mm	1
If the sum of the points is 3 or more, severe calcification should be considered	
IVUS	Points
Superficial calcium angle > 270°and longer than 5 mm	1
360° of superficial calcium	1
Calcified nodule	1
Vessel diameter < 3.5 mm at the calcified lesion	1
If the sum of the points is 3 or more, severe calcification should be considered
- **Step 3:** based on intravascular imaging findings and the calcium scoring system, the operator should select an appropriate plaque-modification device, including non-compliant balloons, cutting or scoring balloons, rotational or orbital atherectomy, or intravascular lithotripsy (IVL). Notably, IVL should only be selected if the calcium score is ≥ 3.
- **Step 4: f**ollowing the initial plaque modification, repeat imaging should be performed to assess the residual calcification. Additional IVL may be used based on lesion morphology. The criteria for IVL use in this context are as follows:
  - In addition to meeting criterion (1), the case must also satisfy either criterion (2) or (3):
    - (1) Post-atherectomy imaging reveals a residual calcium score ≥ 3
    - (2) Further atherectomy is contraindicated due to clinical factors such as reduced left ventricular function or impaired renal function
    - (3) Additional atherectomy is unlikely to be effective based on lesion characteristics
- **Step 5:** once adequate calcium modification has been achieved, further lesion preparation using balloon dilatation may be performed as needed. Final treatment should be accomplished with the implantation of a drug-eluting stent (DES) or, where appropriate, the use of a drug-coated balloon. DES implantation is generally recommended following IVL.

### Assessment of calcification

The severity of coronary calcification should be assessed using intravascular imaging modalities such as IVUS or OCT/OFDI. The angiographic findings are not considered reliable for evaluating calcification severity and are therefore excluded from the assessment criteria. Among various proposed methods, the scoring system presented in Table 1 has been adopted in this consensus document, based on previous reports [3, 4].

## Discussion

Despite recent advancements in device technologies, calcified coronary lesions continue to pose significant challenges during percutaneous coronary intervention (PCI). First, device deliverability may be compromised due to the rigidity of calcified plaque. Second, inadequate stent expansion remains a concern, potentially leading to unfavorable short- and long-term clinical outcomes. Adequate lesion modification prior to stent implantation may mitigate these issues.

Currently available treatment modalities for calcified lesions include non-compliant balloons, cutting or scoring balloons, rotational atherectomy [5], orbital atherectomy, and intravascular lithotripsy (IVL). However, the optimal strategy for device selection and sequencing remains unclear. In the previous consensus document, the concomitant use of atherectomy and IVL was contraindicated. Following the publication of the DUAL-PREP study [2], which demonstrated no safety concerns and superb stent expansion with this combination in the setting of severely calcified lesions, the recommendation has been revised accordingly.

A distinctive feature of the present document is its emphasis on imaging-guided therapy. This approach enables a more tailored and effective strategy for the management of calcified lesions, reflecting the most current evidence and clinical experience. However, current calcium scores are based on stent expansion in the absence of IVL and refer to different findings between IVUS and OCT/OFDI. In addition, there are lesions for which stent placement is not necessarily appropriate, and there are also situations in which it is preferable to end the procedure with DCB. It is necessary to update the guidelines for appropriate use while verifying these clinical questions through clinical trials.

## Data Availability

This article is a consensus document and does not involve the generation or analysis of any datasets.
